# Imaging and management of lymphedema in the era of precision oncology

**DOI:** 10.1093/bjr/tqaf029

**Published:** 2025-02-11

**Authors:** Pranjal Rai, Abhishek Mahajan, Shreya Shukla, Niyati Pokar

**Affiliations:** Department of Radiology, Tata Memorial Hospital, Homi Bhabha National Institute, Mumbai 400012, India; Faculty of Health and Life Sciences, University of Liverpool, L7 8TX Liverpool, United Kingdom; Department of Imaging, The Clatterbridge Cancer Centre NHS Foundation Trust, L7 8YA Liverpool, United Kingdom; Department of Radiology, Tata Memorial Hospital, Homi Bhabha National Institute, Mumbai 400012, India; Department of Radiodiagnosis and Imaging, Mahamana Pandit Madan Mohan Malaviya Cancer Centre and Homi Bhabha Cancer Hospital, Tata Memorial Hospital, Varanasi 221 005, India; Department of Radiology, Tata Memorial Hospital, Homi Bhabha National Institute, Mumbai 400012, India

**Keywords:** lymphatic drainage, lymphatic imaging, lymphedema, lymphedema management, lymphangiography, lymphoscintigraphy

## Abstract

Lymphedema is a common complication of cancer treatment, leading to significant morbidity. Early and accurate diagnosis through the combined expertise of radiology and nuclear medicine is crucial for preventing lymphedema progression and improving patient outcomes. Imaging techniques such as lymphoscintigraphy, duplex ultrasound, MRI, and CT as well as newer modalities including near-infra-red lymphangiography can diagnose and assess lymphedema severity. Bioimpedance spectroscopy provides a non-invasive tool for early detection by measuring extracellular fluid changes, aiding in identifying lymphedema at its earliest stages. Pre-treatment baseline measurements and prospective surveillance models are essential for tracking limb volume changes and mobility, enhancing early intervention outcomes. Recognizing the strengths and limitations of each imaging modality allows radiologists and nuclear medicine physicians to synergistically optimize lymphedema diagnosis and management. Effective management relies on multidisciplinary collaboration and includes conservative and surgical options tailored to disease severity. Advanced imaging modalities are pivotal for planning and monitoring interventional strategies. This review explores the development and management of secondary lymphedema in oncological patients, focusing chiefly on imaging and treatment strategies. It also briefly highlights the evolving role of artificial intelligence and machine learning in enhancing imaging precision and treatment outcomes.

## Introduction

The International Society of Lymphology (ISL) defines lymphedema as a “low-output failure of the lymphatic-vascular system”.^1^ Although less frequent than functional venous insufficiency (commonly referred to as “high-output failure”) or lipedema (abnormal fat accumulation) physicians frequently misdiagnose it as the latter.^2^ Lymphedema might occur as an isolated condition or be accompanied by a variety of other debilitating local consequences (such as fat hypertrophy, inflammation, and fibrosis) or even life-threatening systemic syndromes. Primary lymphedema is less common and its causes include conditions secondary to inherent defects in the lymphatic vessels or lymph nodes such as chromosomal aneuploidies (Klienfelter’s syndrome, Turner’s syndrome) and dysmorphogenic disturbances (Milroy disease, Kippel Treneaunay syndrome).^3^

Secondary causes of lymphedema in surgical oncology include metastasis involving the lymph nodes, surgical block dissections, and radiotherapy.^4^^,^^5^ Being frequently encountered in oncology, understanding lymphedema is paramount for radiologists and nuclear medicine physicians to facilitate early diagnosis and the initiation of preventive strategies. This knowledge is essential to avoid misdiagnoses and unnecessary investigations. Additionally, it enables follow up with patients who have already developed lymphedema to prevent further progression and guides image-directed treatment strategies.^6^ This review focuses specifically on secondary lymphedema in oncological patients, with an emphasis on imaging and its management strategies.

## Lymphedema in oncology

### Aetiologies of secondary lymphedema

While the most common cause of lymphedema worldwide is filariasis,^7^ secondary lymphedema due to malignancy and its treatment tend to be the most frequent cause in developed countries.^8^ Genetics, age, obesity, autoimmunity, and other risk factors have all been linked to the development of lymphedema. Age and obesity are also linked to an increased chance of symptoms worsening in people who already have lymphedema.^9^

The multifactorial aetiology of lymphedema during cancer and its treatment can be grouped into 2 major categories (Supplementary image 1):

1. Causes linked to the primary disease process:
  - Tumour causing compression of lymphatic nodes and vessels, leading to downstream obstruction.
  - Infiltration of lymphatic vessels by the tumour (lymphangitic carcinomatosis).
2. Causes linked to treatment:
  - Surgical dissection of nodes and lymphatics.
  - Radiation effects (secondarily leading to the destruction of lymphatics).
  - Medication effects.

Lymphedema causes are often linked to the timing of disease onset or initial intervention. Lymphedema caused by treatment side effects usually has a better prognosis than when it is caused by the disease itself. Table 1 (modified from Schirger et al.) provides a summary of the causes of lymphedema.^10^ Depending on the time of appearance of lymphedema post-treatment, it can be either early-onset (≤12 months) or late-onset (>12 months). McDuff et al. in their study concluded that axillary lymph node dissection was generally associated with early-onset lymphedema, while radiation therapy was more often seen with late-onset lymphedema.^11^

**Table 1.** Various causes of lymphedema in oncology, their duration and prognosis.

Cause	Duration	Prognosis
Involvement by the disease or compression of the lymphatics by the disease	Permanent and progressive	Poor
Surgical removal of nodes/lymphatics	Usually permanent (may subside with medications and rehabilitative efforts)	Good
Radiation therapy-related effects	Usually permanent	Usually favourable
Medication effects	Usually resolves on cessation of medication or use of alternate medications	Good

The most common cancer associated with the development of lymphedema is breast cancer, with studies reporting 5-year cumulative instances of up to 42%,^7^ usually developing within 2 years of diagnosis.^11^ Other types of cancer include sarcomas, lower extremity melanomas, gynaecologic cancers, and head and neck carcinomas. Head/neck cancers are frequently associated with lymphedema, having an incidence of up to 75%-90% in survivors and can cause difficulties with speech, swallowing, and breathing.^12–14^

The risk of lymphedema is increased as greater number of axillary nodes are removed during breast cancer surgery, and is more common post-mastectomy as compared to wide local excision. A higher body mass index (particularly >25-30) and adjuvant radiation therapy are also linked to a higher risk in post-lymphadenectomy patients.^1^^,^^9^^,^^15^^,^^16^

Generalized, insidious, and progressive limb swelling which usually demonstrates pitting oedema in the early stages, and becomes non-pitting in later stages due to cutaneous fibrosis and adipose tissue deposition is a characteristic feature. The “Stemmer sign” which is the thickening of skin fold at the second toe or finger has a high positive predictive value for diagnosing lymphedema.^17^ Ulceration and recurrent infections may occur in later stages.

### Staging systems and assessment methods

The International Society of Lymphology (Table 2) categorizes lymphedema into distinct stages. This staging system primarily focuses on the diagnosis and progression of lymphedema, employing a qualitative approach. Within each stage, a limited but nonetheless functional severity assessment (quantitative approach) has been used. This assessment uses simple excess volume differences to categorize the severity as minimal (>5% increase in limb volume), moderate (20%-40% increase), or severe (>40% increase).^1^

**Table 2.** Lymphedema stages as described by the International Society of Lymphology (ISL).

ISL stage	Description
0	A subclinical state where swelling is not evident despite impaired lymphatic transport. This stage may exist for months or years before lymphedema becomes evident
1	Early onset disease stage where there is an accumulation of tissue fluid which subsides with limb elevation. The oedema may be pitting
2	Limb elevation rarely alone reduces swelling and oedema is pitting
Late 2	Tissue fibrosis is more evident and pitting is reduced
3	Tissue is hard and fibrosis has set in. Pitting is absent, with evidence of tissue hyperpigmentation, fibrosis, and adipose tissue hypertrophy

The National Cancer Institute’s Common Terminology Criteria for Adverse Events (CTCAE) is used to stage lymphedema in research and clinical settings. It relies on patient reported physical impediments rather than objective measurements, making it less reliable for assessing true lower extremity lymphedema.^8^

Other classification systems include the Campisi scale, imaging based systems such as Taiwan Lymphoscintigraphy Staging (TLS) and Indocyanine Green (ICG) Lymphography Staging and Cheng’s Grading System.^6^

In patients undergoing cancer treatment, monitoring for the development of lymphedema has become paramount for the early identification, leading to conservative interventions, greater treatment success, and potential cost reductions. This can be achieved through the utilization of prospective surveillance models, which establish pre-treatment/pre-operative baseline measurements for both limbs, including volumes and mobility assessments.^9^ The utilization of objective measures, such as Tissue Dielectric Constant and Bioimpedance Spectroscopy (BIS), demonstrates even greater efficacy in the early identification of lymphedema (especially in ISL stage 1) with few studies even demonstrating level I evidence.^18–20^

BIS is a non-invasive tool that determines the quantity of extracellular fluid by measuring tissue resistance to the flow of electric current, thus indirectly assessing lymphedema. The ImpediMed L-Dex U400 was the first FDA approved BIS device used in the United States. An L-Dex score of more than 10 is diagnostic of lymphedema.^21^

Other measurement techniques include Water displacement volumetry (which includes submerging the affected limb in water and calculating the amount of volume displaced) and Perometry (which involves projecting infrared light onto the affected limb and capturing the reflected light using sensors).^22^

Simpler assessment methods, such as measuring limb circumference using the 4-point system or calculating limb volume, also aid in diagnosis, especially in resource-limited settings. A volume increase of ≥10% or limb circumference increase ≥2 cm is typically defined as diagnostic criteria for lymphedema.^9^^,^^23^ However, a comprehensive clinical and treatment history, along with the clinical features, usually suffice for diagnosis.

## Imaging of lymphedema

### Lymphoscintigraphy

This minimally invasive technique involves intradermal injection of radiopharmaceuticals (such as ^99m^Tc nanocolloid albumin, sulfur colloid, phytate, or antimony sulfide) into the interdigital space of both the feet, followed by subsequent detection of the gamma photons with the help of a dedicated dual detector high-resolution gamma camera with parallel hole collimators in the whole-body scanning mode (at 20 min and 3 h)^24^ and currently holds level I evidence for investigation of suspected lymphedema.^1^ The principle is initial uptake by dermal lymphatics, which flows into the lymphatic system, and then downstream into the nodes. The lymphatic system is driven by a combination of external osmotic and oncotic forces, smooth muscle within the vessel walls, and decentralized muscle contractions throughout the body.^25^ The optimal particle size for radiopharmaceutical is generally considered to be between 50 and 70 nm. These particles typically enter the lymphatic system but are too large to pass through the blood capillaries. Conversely, smaller particles (<15 nm) rapidly migrate and saturate the clearing capacity of the first draining lymph node. Although this may not be particularly useful for detecting sentinel lymph nodes, it can aid in visualizing more lymph node tiers, which is particularly helpful in lymphoscintigraphy (LS) of the extremities.^6^ The protocol for LS varies among diagnostic centres, depending on the choice of radiopharmaceutical used, the injection type and site, the use of dynamic or static acquisitions, and lastly the acquisition times.^26^

Advantages of LS include depiction of lymphatic anatomy as well as assessment of lymphatic flow disorders. It provides both qualitative and quantitative information related to the lymphatic system. Qualitative information includes lymphatic system morphology and course, number of lymph nodes, lymphatic vessel asymmetry, and the presence of dermal backflow. Dermal backflow is demonstrated by reflux from the collecting lymphatics through the pre-collecting lymphatic channels into the initial superficial dermal collecting lymphatics. This occurs secondary to an upstream increase in pressure gradients. Quantitative information encompasses the measurement of uptake and clearance times from the time of radiopharmaceutical injection.^27^ However, its practical application is limited due to the time-consuming nature of obtaining these measurements and the inconsistency of the results obtained. Furthermore, the definitive diagnosis often relies on qualitative measurements only.^6^

**Figure 1.**
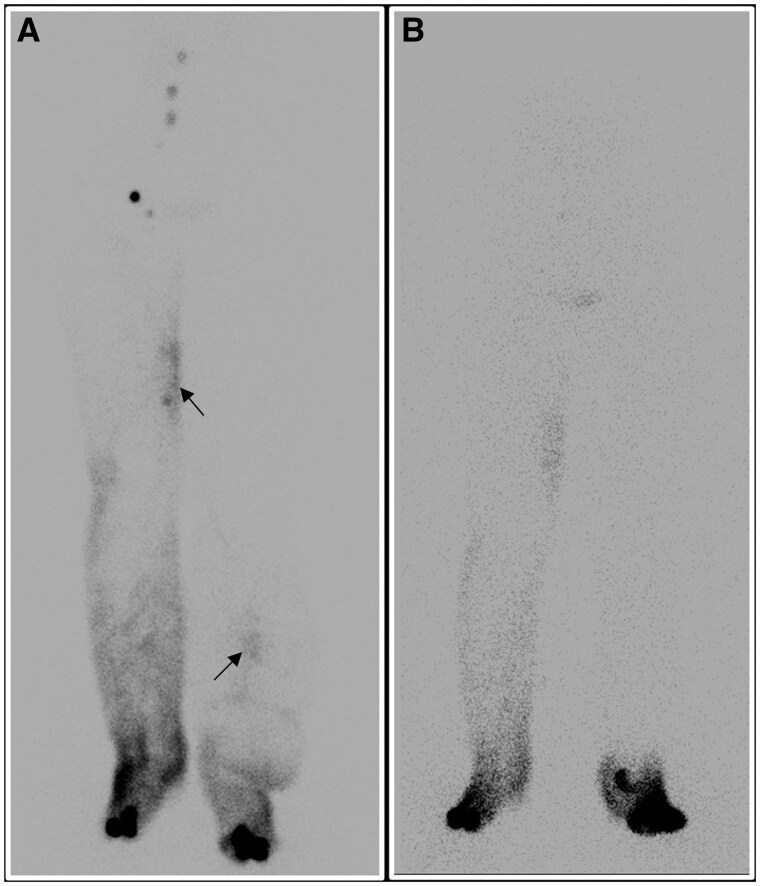
^99m^Tc nanocolloid lymphoscintigraphy done in a patient of carcinoma cervix with bilateral pedal oedema, (A) shows prompt visualization of right inguinal and pelvic lymph nodes, with significant dermal backflow in both the lower limbs (arrows). The patient underwent lymph node transfer surgery, however, 10 years post-surgery, the patient presented with a raw area over the dorsum of the foot. A repeat lymphoscintigraphy scan (B) was done which did not demonstrate the transferred lymph node in the left lower limb suggesting the non-viability of the lymph node.

LS also demonstrates utility in the assessment of post-therapeutic results (Figure 1).

Subfascial injection of the radiopharmaceutical can be used for assessment of the deep lymphatic drainage. Thus, the injection site (intradermal, subcutaneous, or subfascial) depends on the scope of the study and the type of radiopharmaceutical used. The total recommended activity to be administered in adult patients is 74 MBq, which is equivalent to 37 MBq per limb and per investigated compartment. This amount can be administered in single or multiple aliquots, depending on the patient’s individual needs and the specific anatomical area to be examined. The injected volume per aliquot should not exceed 0.2 mL. In contrast, paediatric patients require a lower activity level, with a recommended range of 0.5-1 MBq/kg body weight per limb. This amount should be administered in a single injection, with a maximum volume of 0.2 mL.^26^

The Taiwan Lymphoscintigraphy Staging is a consistent and reproducible scale that is based on the uptake of the proximal lymph nodes at 120 min for diagnosing lymphatic disorders. There are 3 groups and 7 stages, based on the visualization of proximal lymph nodes, distal lymph nodes, the lymphatic ducts, and the presence or absence of dermal backflow (Table 3). While being reliable for diagnosing the severity of lymphedema, it is not recommended in every single patient with extremity oedema but rather in patients with a history of cancer treatment in the form of lymph node dissection or radiation therapy.^28^

**Table 3.** New Taiwan lymphoscintigraphy staging for unilateral extremity lymphedema.^28^

Category	Normal lymphatic drainage	Partial obstruction			Total obstruction		
Stage	L-0	P-1	P-2	P-3	T-4	T-5	T-6
Proximal lymph nodes	+	+/↓	↓	−	−	−	−
Intermediate lymph nodes	−	−	+/−	+	−	−	−
Lymphatic ducts	+	+/Distal	Distal/Engorged	−	Engorged/−	Engorged/−	−
Dermal backflows	−	−	+(Proximal/Distal)	+(Distal/Entire)	+(Distal)	+(Entire)	−

Proximal lymph node symbol meaning. “+”, “↓”, and “−”: good visualization, reduced visualization, and absent visualization of proximal lymph nodes, respectively.

Reduced or absent visualization means reduced or no visualization of the proximal lymph nodes compared to the contralateral healthy limb.

Intermediate lymph node symbol meaning. “+”, “−”: presence or absence, respectively, of lymph node uptake at the level of the elbow or knee.

Lymphatic duct symbol meaning: “+”: visualization of the tracer in the lymphatic ducts of all limbs, “Distal”: visualization of the tracer only in the lymphatic ducts of the distal limb due to incomplete uptake; “Engorged”: abnormal radiotracer accumulation or extravasation; “−” no visualization of lymphatic ducts.

Dermal backflow (spread of lymph fluid through the dermis due to obstruction of proximal lymph nodes or injured lymphatic ducts) has been classified into 3 types: “Proximal” in the case the skin pattern is seen up to the knee or elbow level; “Distal” in the case of visualization below the knee or elbow joint; and “Entire” when the skin pattern was present throughout the limb.

LS has high sensitivity (up to 96%) and a very high specificity (up to 100%) for diagnosing lymphedema.^29^ Clinical applicability of LS extends to evaluating lymphatic anatomy and function, identifying “at-risk” regions, tailoring treatment strategies (by distinguishing the extent of involvement in the affected area, guiding treatment modality, dosage, and frequency, and specifying direct compression requirements), diagnosis (differentiating from other oedema aetiologies and assessing focality), and monitoring treatment efficacy (particularly changes in lymphatic function).^24^

Disadvantages of LS include the lack of standardization regarding the employed radiopharmaceutical, injection site, dosages, imaging timings, quantitative analysis of results, poorer sensitivity in detecting ISL grade I lymphedema when compared to other modalities,^30^ and poor spatial resolution.^24^ There is no direct information obtained regarding precise vessel calibre. Notably, the images acquired in this technique are planar, ie, they do not contain any depth information and suffer from overlap.^31^ This can be alleviated with the use of single photon emission CT (SPECT).

Yoon et al investigated the potential advantages of SPECT/CT compared to planar LS for the initial staging of secondary extremity lymphedema. Their study revealed a modification rate of 15.4% in lymphoscintigraphy staging (TLS). Limitations of planar LS, including missed dermal backflow, imprecise lymph node uptake assessment, and misregistration due to skin or clothing contamination, were overcome by combining SPECT with anatomical CT data. This combined approach facilitates more accurate lymphedema staging.^32^ Similar findings were also reported by Weiss et al in a related study, wherein the addition of SPECT/CT, as opposed to planar scintigraphy, provided pertinent information regarding the presence of dermal backflow (86%), the anatomical extent of lymphatic disorders (64%), the presence or absence of lymph nodes (46%), and the visualization of lymphatic vessels (4%).^33^

While SPECT/CT offers improved diagnostic accuracy, it is crucial to consider the increased radiation dose and cost compared to planar LS. Additionally, lymphoscintigraphic staging may not always translate to changes in treatment, particularly surgery. Its primary value lies in monitoring disease progression and informing management strategies for lymphedema.^34^

### Near-infrared lymphangiography

This is one of the more recent and promising lymphedema diagnosis methods based on utilizing fluorophores like ICG. Fluorophores are photosensitive chemical compounds that get excited when exposed to a near-infrared (NIR) range of light, thus generating fluorescent signals. These signals are captured with the help of a dedicated camera and used to construct images of tissue containing the imaging agents.^6^ This method was initially used for sentinel node mapping, and now helps stage lymphedema and assess surgical eligibility.^24^

Its superior spatial and temporal resolution enables visualization of the lymphatic vessel anatomy and lymphatic transport problems that may be seen in both pre-clinical and clinical settings of lymphedema.^35^^,^^36^ More noticeable signs such as dermal backflow, seen in later stages of lymphedema, are also better demonstrated.^37^ Additional benefits include better affordability and easier executability.^38^

The depth limitation of NIR-based imaging, which ranges from 2 to 4 cm, prevents its applicability in obese patients.^39^ Such cases require a hybrid approach, pairing multiple imaging modalities like LS with ICG imaging.^40^

Both traditional LS and NIR-based imaging techniques have been utilized for planning surgical treatments of lymphedema such as lymphatic venous anastomosis. They can precisely assist in identifying the incision site, assess lymphedema before and after surgery, and even provide intraoperative guidance.^41^

This technique is highly sensitive (100%) and specific (100%) for detection of lymphedema.^28^ The clinical applicability of NIR-based imaging encompasses the assessment of lymphatic anatomy and function. It serves as a screening tool to identify “at-risk” territories, detect early changes, and facilitate longitudinal evaluation, all of which can be performed independently of a normal comparator. Furthermore, NIR-based imaging enables personalized treatment planning, diagnosis, and monitoring of treatment response, similar to the capabilities of LS.^24^

### Lymphography

This technique was developed in the late 50s, by Professor John Kinmonth, and was based on the principle of cannulation of superficial dermal lymphatics and injecting an oil-soluble iodinated contrast (ultra-fluid lipiodol) with the help of a 30-gauge needle under an operating microscope.^42^ Modern modification of this technique includes indirectly targeting the lymphatic vessels by injecting a water-soluble iodinated contrast intradermally, followed by its uptake into dermal lymphatics. X-ray imaging is then performed (fluoroscopy, radiography, or CT) to visualize the contrast material. It helps in imaging lymphatic vessels, and backflow of contrast in cases of primary and secondary lymphedema. This technique circumvents the morbidity associated with the conventional lymphography, and because of the excellent penetration, allows visualization of deep lymphatic structures, such as the thoracic duct. It can also identify lymphatic leakage at different sites which may occur secondary to an iatrogenic cause, thus also providing an opportunity for embolization.^37^ Its main disadvantage is its inability to provide sufficient contrast over large fields of view thus limiting its clinical applicability.

### Ultrasound imaging

Duplex ultrasound (DUS) is a widely available, cheap, and non-invasive imaging modality that has traditionally been used in the assessment of venous obstruction with associated limb swelling with or without an additional lymphatic abnormality. The indirect assessment of the tissue layers (the epidermis, dermis, and subcutaneous layer) can offer information regarding the cause of obstruction. Imaging findings include skin thickening, water accumulation, and stone-paved appearance. The presence of stone-paved appearance and hypoechoic areas in the subcutaneous space is related to the percentage increase of excess volume (PEV) secondary to the subcutaneous fluid accumulation causing thickening of the subcutaneous tissue layer. This imaging finding may further serve as a marker for predicting better treatment outcomes in patients undergoing compression therapy. Hyperechoic appearance on the other hand, representing interlobular and intralobular water accumulation and/or interlobular and intralobular fibrosis, may have worse outcomes.^43^

**Figure 2.**
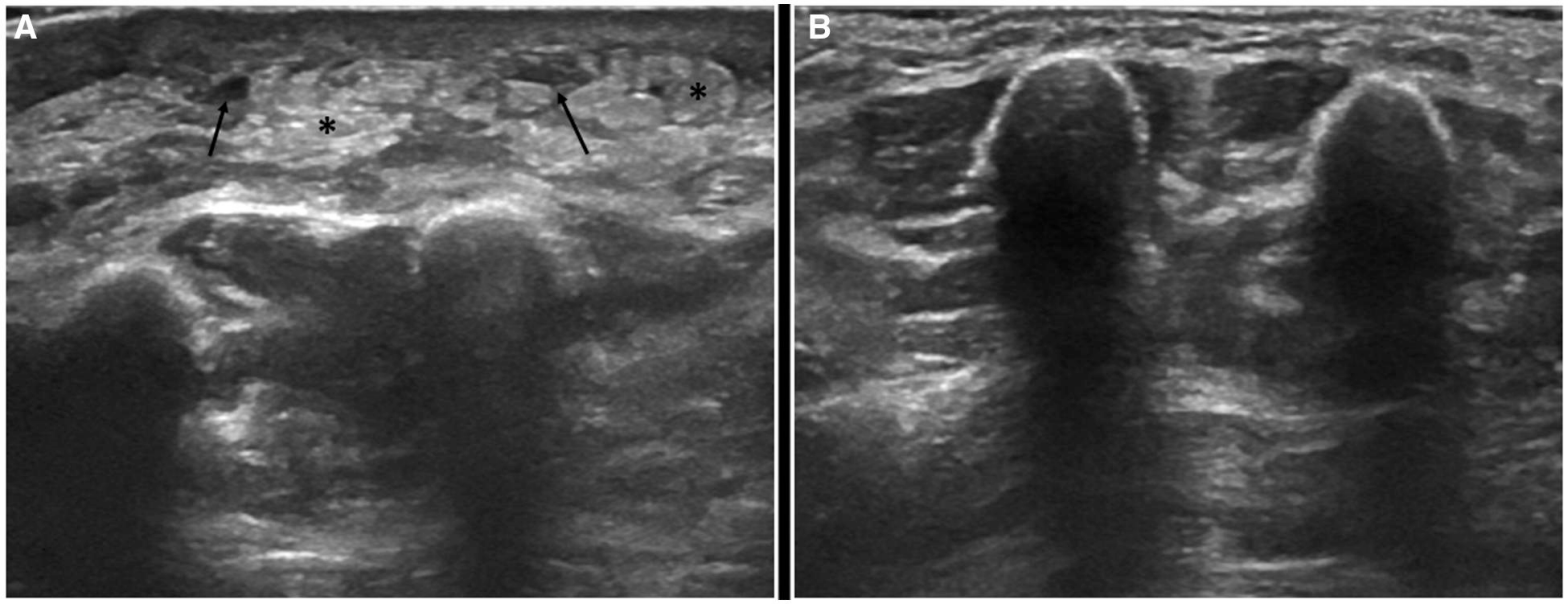
Ultrasound of the right limb (A) at the mid-forearm level in a patient operated for right breast cancer, post radiation therapy, 6 months back, with increased hyperechogenicity and hypertrophy of the subcutaneous fat (asterisk), with associated interspersed fluid clefts in between the fat lobules (arrows). Findings are consistent with grade 1 SEG. Note the normal thickness of the dermis as well as the subcutaneous tissue in the contralateral limb (B).

DUS can also help correlate the grade of lymphedema (subcutaneous echogenicity grade or SEG) with the ISL lymphedema staging system as demonstrated by Suehiro and colleagues in their study.^44^ Cellular changes such as hypertrophy of the connective tissue, increase in the number of fat globules, and build-up of interstitial fluid within the subcutaneous layer were used to predict the lymphedema stage (Figure 2) (Supplementary Image 2). They showed that the thickness of the skin and the subcutaneous tissue, as well as the increase in echogenicity of the subcutaneous tissue, correlated well with the ISL staging. Usually, it is recommended that lower limb ultrasound imaging be assessed at the level of mid-calf, and upper limb imaging be assessed at the level of mid-arm.^45^

Recently, ultrasound elastography (UE) has also been used as an objective tool for indirectly assessing subcutaneous fluid accumulation by the assessment of tissue elasticity.^46^ Utilizing a transducer in B-mode, UE is a quasi-static approach that compresses the tissue and then uses the difference between the compressed and reference images to derive an image of the strain that is created. This computation, which may qualitatively demonstrate tissue stiffness in a colour image, is generated with the aid of a 2D correlation of traditional ultrasound scans (B-mode images).^47^ In the early stages of lymphedema (stages 0, 1, and 2), the presence of water within the subcutaneous plane leads to increased strain and deformity, and as the severity of lymphedema increases, gradual subcutaneous tissue fibrosis results in hardening (Supplementary Image 3). Operator dependence and limited assessment depth are the limiting factors. Additionally, standard thresholds for diagnosis and surveillance are not established.^36^

In clinical practice, DUS allows assessment of tissue composition (fluid component), allowing assessment of “at-risk” territory, distinguishing affected and unaffected areas and individualizing treatment options (similar to LS and NIR lymphangiography).^24^

Recent research has shown a growing interest in the potential application of low-intensity pulsed ultrasound (LIPUS) for reducing lymphedema. The therapeutic effect of LIPUS in lymphedema is hypothesized to stem from its ability to modulate inflammatory processes. LIPUS vibrations are thought to promote cell and tissue movement, potentially loosening and disrupting lesions. Furthermore, LIPUS may enhance tissue metabolism and circulation,^48^ potentially improving nutrient delivery and reducing overall inflammation.^49^ Additionally, studies suggest LIPUS may directly regulate macrophage polarization, reducing the pro-inflammatory M1 phenotype and promoting an anti-inflammatory M2 phenotype.^50^ These findings suggest a potential therapeutic role for ultrasound, expanding its utility beyond its well-established application in diagnostic imaging.

### Computed tomography

**Figure 3.**
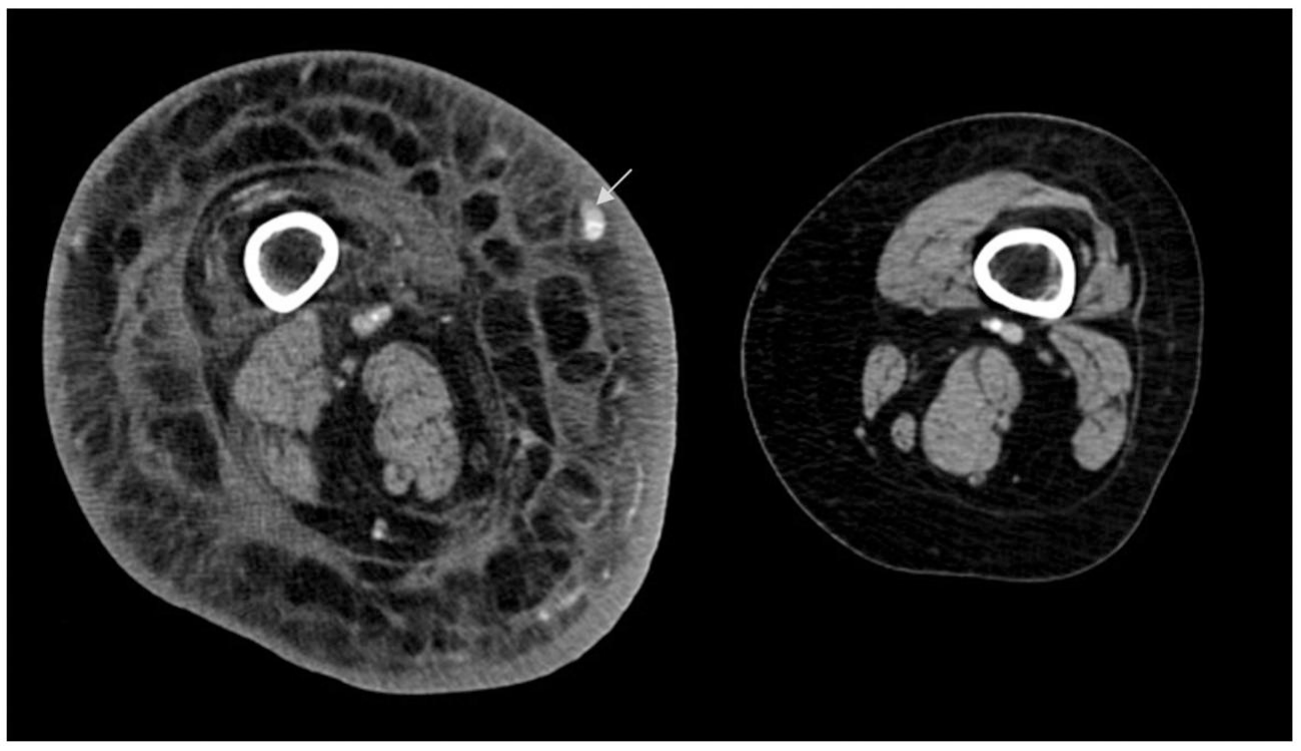
Contrast-enhanced CT scan of bilateral lower limbs of a patient with a right thigh synovial sarcoma, who developed swelling in the right lower limb, a year after surgery and radiotherapy. Note the diffuse skin thickening and cobblestone appearance of the subfascial and epifascial plane, due to the fluid and the fibrosis surrounding the fatty tissue. Prominent venous collaterals are also seen in the subcutaneous plane (arrow). The contralateral limb appears normal.

CT studies help detect lymphedema and localize it to the subfascial or epifascial plane. The pathognomonic honeycombing seen on CT imaging is secondary to the fluid surrounding the adipose tissue and the fibrosis, and is not found in conditions such as lipodystrophy and oedema of venous origin (Figure 3).^32^ Fluid lakes suggesting dermal collateral lymphangiectasis may also be encountered occasionally, along with associated thickening of the skin and the absence of oedema within the muscular compartments.^51^ CT is more helpful in the detection of causes of secondary lymphedema and can also show pathologically enlarged lymph nodes. Studies have compared CT and LS, where CT has emerged superior in terms of both sensitivity (93%) and specificity (100%).^24^ This technique is avoided owing to the ionizing radiation.

Clinical applicability of CT stems from its ability to assess volume, dysmorphism, tissue composition (fluid content), and metaplasia. Although it lacks significant value for screening purposes, CT enables personalized treatment options by distinguishing the extent of involvement in the affected region and allowing the differentiation between affected and unaffected areas. Although not commonly employed for this specific reason, CT can also serve as a monitoring tool due to its sensitivity to alterations in tissue composition.^24^

Perhaps the major utility of CT for lymphedema can be better explored in terms of its combined use with SPECT, as discussed in the prior section.

### MRI and MR lymphography

Both non-contrast and contrast-enhanced MRIs are useful for the assessment of peripheral lymphedema.

Lymph has an inherently high T2 relaxation time (∼600 ms at 3.0 T),^52^ necessitating the use of heavily T2-weighted sequences such as the Short-tau inversion recovery (STIR) and T2-fat-suppressed sequences to visualize the dilated lymphatic channels and to characterize oedema patterns in NCMRL. Extremity imaging is usually performed for both limbs together, allowing for comparison with the unaffected side. “Superman position” is usually employed for imaging upper limbs where the affected limb is extended above the patient’s head and the unaffected limb is placed along the patient’s side.^53^ In addition to pre-surgical planning, non-contrast MR lymphography (NCMRL) can be used for post-surgical assessment of oedema following lymph node transplant. NCMRL helps in the detection of dilated vessels, which is pronounced in primary lymphedema but cannot differentiate lymphatics from superficial veins. Literature reports the sensitivity and specificity of MR lymphangiography of 100%.^54^

Axial acquisitions allow assessment of the degree of oedema and distribution while 3D coronal acquisitions are more efficient in covering large anatomic areas. STIR sequences correlate well with the degree of clinical lymphedema where classical imaging patterns such as epifascial fluid, honeycombing, and dermal thickening are well visualized.^55^

**Figure 4.**
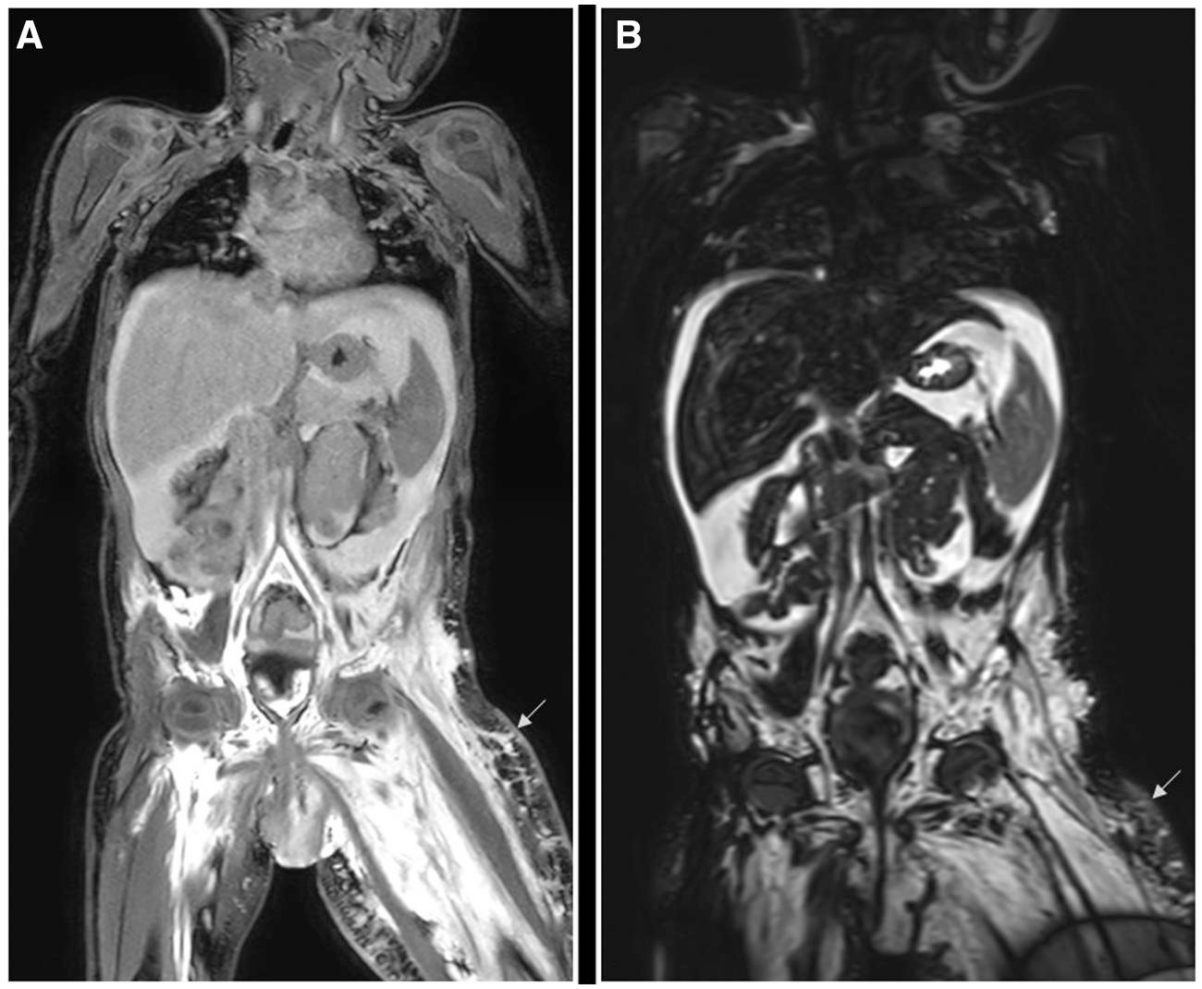
Magnetic resonance lymphangiogram performed at 1.5 T Philips Ingenia, (A) shows 3D-T1-weighted DIXON image acquired after 20 min post-injection of 10 mL gadobenate dimeglumine intracutaneous in bilateral lower limbs and (B) showing coronal VISTA (3D volume isotropic TSE sequence) acquisition in the same patient with a bilateral lower limb lymphovascular malformation, showing extensive dermal backflow, more on the left side (arrows). There is significant oedema in bilateral lower limbs and the vulva region with associated fat stranding and fibrotic changes suggestive of chronic lymphatic obstruction. VISTA: Volume ISotropic Turbo spin echo Acquisition.

A large amount of oedema can obscure the underlying fat hypertrophy on traditional T2-weighted fat-suppressed images, however, the use of Dixon fat-separating techniques can alleviate this problem. Ancillary features of lymphedema that can be appreciated on NCMRL include fat deposition and secondary muscle atrophy due to decreased muscle activity. The inability to visualize dynamic processes such as active chyle leaks and dermal backflows limits the use of NCMRL. Another disadvantage is the poor resolution of the 3D-gradient echo-based sequences. These issues are alleviated with the use of contrast-enhanced MR lymphangiography (CE-MRL) (Figure 4).

CE-MRL uses an injection of gadolinium-based contrast agents such as gadopentetate dimeglumine (Magnevist, Gd-DTPA) or gadobutrol (Gadaist, Gd-DTPA-BMA) admixed with saline forming a 1:1 isotonic solution. The solution is generally injected through the intradermal route or intranodally.^56^ The injection volume is generally 0.5-2 mL with greater injection volumes generally being preserved for the intranodal route.

After contrast injection, visualization can be performed with the use of fat-saturated T1-spoiled gradient recalled echo (SPGR) based sequences.^57^ This technique provides better spatial resolution on the order of ∼1 mm^3^, however, one challenge associated with it is venous contamination. Dermal contrast being taken up by both lymphatics and venous systems can make the distinction between them difficult.^56^ However, venous features such as their larger calibre, smoother walls, and lesser tortuosity can help distinguish the 2.

Clinical applicability of MRL lies in its ability to assess volume, dysmorphism, tissue composition (fluid content), metaplasia, and lymphatic anatomy. Like CT, MRL is helpful in screening due to its sensitivity to early changes. Distinguishing degree of involvement in the affected category, it also helps individualize treatment while also identifying contributing factors. Its sensitivity to change in volume and tissue composition also allows for treatment monitoring.^24^

### Newer imaging techniques

Advancements in MRI techniques for lymphatic imaging in the form of the use of gadolinium-chelated nanoparticles as shown by Müller et al have allowed for excellent visualization of lymphatic anatomy in healthy animals, with high signal-to-noise ratio. However, the clinical application of these techniques remains distant.^58^

The assessment of lymphedema cannot fully rely on Positron emission tomography (PET) because of its low spatial resolution, high equipment expenses, and radioisotope usage. Its excellent depth penetration and high sensitivity, however, have enabled it to be utilized in many experimental studies. Hou et al. used ^68^Ga-NOTA-Evans Blue TOF PET/MR LS to evaluate the severity of lower limb lymphedema, which provided excellent visualization of the position and depth of the lymphatic vessels, thus helping guide microsurgical techniques.^59^ Another study by Long et al. yielded comparable outcomes.^60^ There have also been studies utilizing PET in conjunction with CT and MRI for 3D visualization of draining lymph nodes in animal models, where ^18^F-FDG was used as a tracer.^61^^,^^62^

Recent advances in preclinical imaging of the lymphatic system have put fluorescence imaging at the forefront because of its lack of radiation, better resolution, and affordability. The technique utilizes fluorescent dextrans which enter the lymphatics and thus allow visualization of the lymphatic-vascular morphology with the help of multiphoton microscopy. This has been successfully implemented in various murine models.^63^ Photoacoustic imaging is a form of hybrid imaging technique where the tissue of interest is illuminated with short-pulsed non-ionizing light. The illuminated tissue undergoes subsequent thermoelastic expansion due to the heat generated. Thermoelastic expansion leads to the production of ultrasound waves which are detected with the help of transducers, following which a post-processed image is generated.^37^ This technique has shown tremendous promise owing to its high scalability and better resolution at greater depths as compared to optical imaging.^64^

### Artificial intelligence and future promises

Advancements in machine learning bridge software, healthcare, and computer sectors have also fuelled artificial intelligence (AI) and radiomics applications in lymphedema identification.

In their study, Nowak et al showed the utility of a deep learning (DL) model for analysing lower extremity MRIs of patients suffering from lymphedema.^64^ Son et al have provided an early example of a DL-based algorithm's application potential in the CT-based identification of lymphedema-induced fibrosis in their study.^65^ Studies have also been done to achieve the reproducibility of DL algorithms in operator-dependent models such as ultrasonography, as demonstrated by Goudarzi et al.^66^

There is critical need for standardization of imaging protocols and acquisition techniques to allow for increased reliability of AI systems and to enable more robust analysis.^24^^,^^31^

It is predicted that, with the exponential expansion of AI, these DL-based algorithms will help physicians diagnose lymphedema and provide timely treatment alternatives.

## Management of lymphedema

### Primary prevention

Primary prevention techniques aim to prevent lymphedema from developing and enhance patient well-being. In patients with breast cancer, this is achieved by opting for axillary lymph node biopsy for staging instead of axillary node dissection.^67^ Furthermore, evaluating the risk factors on an individualized basis before surgery and radiotherapy can help the clinician to expect lymphedema in the immediate post-treatment period, and hence early treatment strategies can be utilized before late-stage lymphedema sets in. Limiting lymph node dissection is the only method that has been known to demonstrate a reduction in the incidence of lymphedema in the postoperative period. Other surgical techniques such as reverse mapping and the use of lymphatic bypass are helpful when lymph node dissection is necessary.^68^ Limiting the radiation with the use of advanced radiation techniques may be helpful. Range-of-motion exercises and compression sleeves are advised for the afflicted arm following breast cancer therapy.^69^ Exercise and weight training are also encouraged once the surgical wounds have healed. Exercise, particularly aerobic and resistance training, should be done while wearing properly fitted compression clothing.^70^ Lymph node dissection can also be combined with lymphovenous bypass, or instant lymphatic reconstruction (ILR), to assist prevent lymphedema.^71^

### Secondary prevention

Lymphedema management begins with compression therapy, which is frequently supplemented with physiotherapy in the form of manual lymphatic drainage (MLD).^5^ Compression therapy involves the use of short-stretch bandages and multilayered padding materials. Fitted compression clothing (at least class I compression) worn while awake is used in maintenance therapy to prevent reaccumulation of lymphatic fluid. To prevent hand swelling when wearing a compression sleeve, a compression handpiece, like a glove or a gauntlet, is required. Compression clothing can reduce swelling when properly fitted and worn; however, improperly fitted clothing can be constrictive and can worsen lymphedema.^72^ Garments should be replaced every 6 months because they lose elasticity.

In certain subgroups of patients, particularly those with breast cancer-associated lymphedema, MLD, especially for those with mild-to-moderate lymphedema, appears to provide an extra advantage to compression therapy in reducing swelling.^73^ MLD is a massage-like method used by specially trained physiotherapists. Studies have demonstrated that MLD enhances emotional function, and reduces the occurrence of dyspnoea, and sleep difficulties while improving other aspects of quality of life.^74^

To prevent recurrent infections, complex decongestive therapy is a 2-phase, complete physical therapy that includes exercise, daily compressive bandaging, MLD, and skin care with nail care. It is a time-consuming and expensive option that needs lifelong maintenance therapy. An intermittent pneumatic compression pump utilizes a stocking that is intermittently inflated over the arm, in a sequential manner from a distal to proximal fashion. It is applied 4 to 5 times per week, daily.^62^

Initially, the aforementioned non-operative methods are the primary approaches for treating lymphedema. Surgical intervention becomes necessary in cases of secondary lymphedema under various circumstances, including localized primary lesions, unsuccessful non-operative treatment, recurrent cellulitis, lymph leakage, functional limitations, deformity or disfigurement, pain, and diminished quality of life. The objectives of surgical management are to alleviate pain and discomfort, restore or preserve functionality, minimize infection risk, prevent disease progression, enhance appearance, and mitigate deformity. The timing of surgery and specific surgical procedures are not universally agreed upon, and the decision to proceed with surgery should be based on individual considerations. A pre-operative evaluation is necessary to confirm the aetiology of lymphedema, which is also intended to rule out lymphedema related to chronic conditions such as heart failure or protein deficiency. The 3 important things that need to be evaluated include the degree of lymphedema (measured using the arm width) and the grade of lymphedema according to ISL. A traditional duplex ultrasound is recommended to rule out deep venous thrombosis, venous insufficiency, and valvular incompetence.^71^ Two broad techniques in the management of secondary lymphedema are physiologic and reductive.

Physiologic treatments involve lymph node transplantation and lymphaticovenous anastomosis or LVA. In lymph node transplantation, healthy lymph nodes are obtained from a donor site and surgically transplanted to the affected limb with reattachment of arterial and venous circulation. In microsurgical techniques like LVA, lymphatic vessels are anastomosed with surrounding venous structures, thus allowing lymph to re-enter the circulation and preventing its build-up in the interstitial space. These procedures are employed for patients with early lymphedema before fibrosis settles in.

Reductive techniques aim to eliminate the accumulated fibrofatty tissue. These involve either liposuction, where small cannulas remove the subcutaneous tissue, or, less frequently, radical resection of the excess tissues. These are most suitable for patients unresponsive to conservative treatments or having advanced lymphedema with fat deposits and fibrosis.

## Conclusion

Lymphedema, a chronic accumulation of lymphatic fluid in soft tissue poses a significant complication for cancer patients undergoing surgery and/or radiation therapy. This review focused on secondary lymphedema in this population, emphasizing imaging and management strategies.

Understanding the role of various imaging modalities, their importance and limitations, and their synergy with diverse therapeutic options is crucial for effectively addressing secondary lymphedema in oncological patients. Ongoing research and development in both imaging and management hold promise for improving the quality of life for these individuals.

## Supplementary Material

tqaf029_Supplementary_Data
