# Supplementary material for: Imaging and management of lymphedema in the era of precision oncology
Source: Br J Radiol. 2025 Feb 11;98(1169):619–29. doi: 10.1093/bjr/tqaf029 (PMC12012379; doi:10.1093/bjr/tqaf029)
Supplement: tqaf029_Supplementary_Data [file tqaf029_supplementary_data.zip › tqaf029_Supplementary_Data/Supplementary image with legends.docx]

**Supplementary figures:**

**
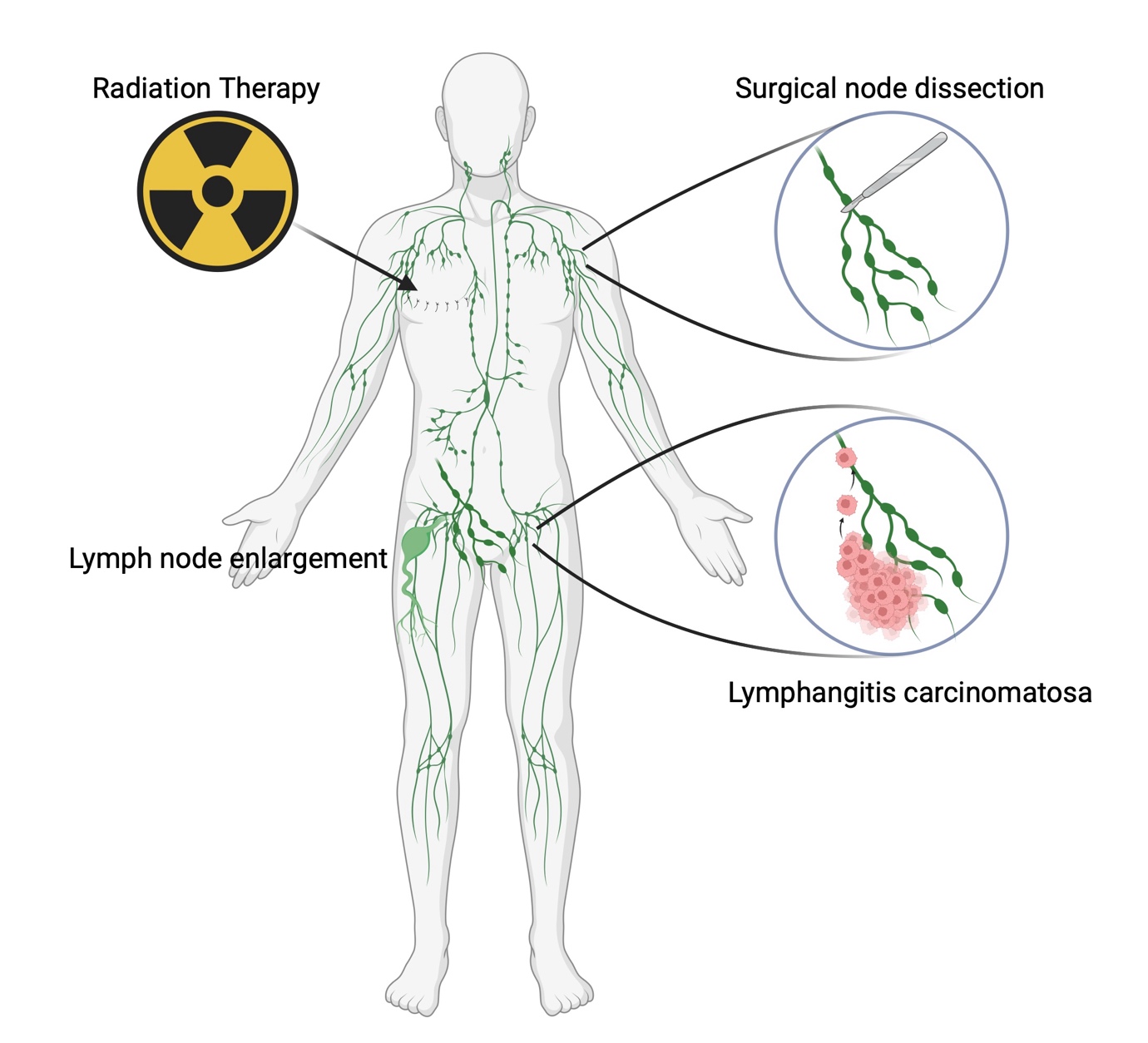
**

Supplementary Figure 1: This figure shows secondary lymphedema in cancer, caused by treatment effects (lymph node dissection, radiation therapy) or the disease process itself (enlarged nodes causing downstream obstruction, lymphatic vessel invasion). Created with BioRender.


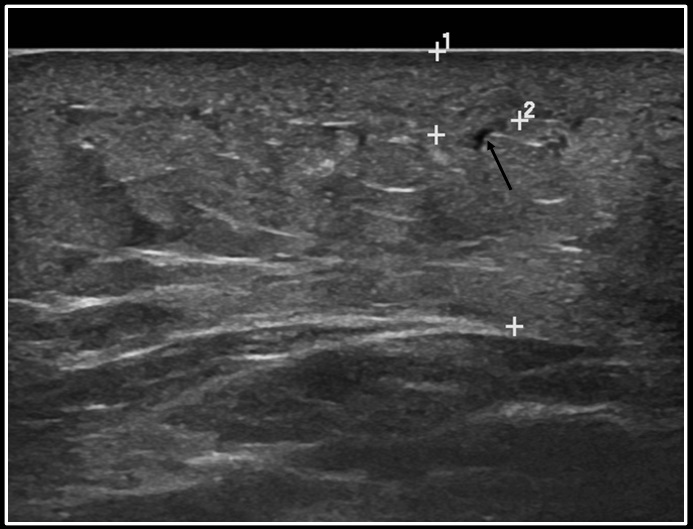


Supplementary Figure 2: Elastography of the affected limb (A) in a patient with lymphedema, with color-coded maps showing an increase in the hardness of the affected soft tissue (blue areas). Contralateral limb (B) shows normal hardness of the soft tissue with uniform distribution of the color map.


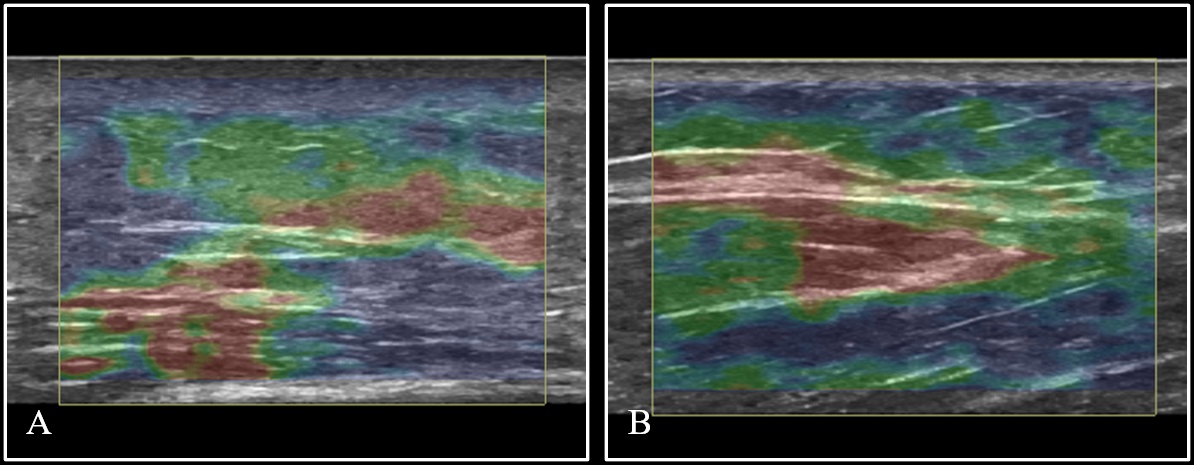


Supplementary Figure 3: Greyscale ultrasound of a patient with lymphedema, with the increase in thickness of dermis (1) and subcutaneous fat (2), with minimal fluid clefts (arrow) in between the areas subcutaneous fat hypertrophy, consistent with grade 1-2 SEG.
