# Supplementary figures and images for: Imaging and management of lymphedema in the era of precision oncology
Source: Br J Radiol. 2025 Feb 11;98(1169):619–29. doi: 10.1093/bjr/tqaf029 (PMC12012379; doi:10.1093/bjr/tqaf029)

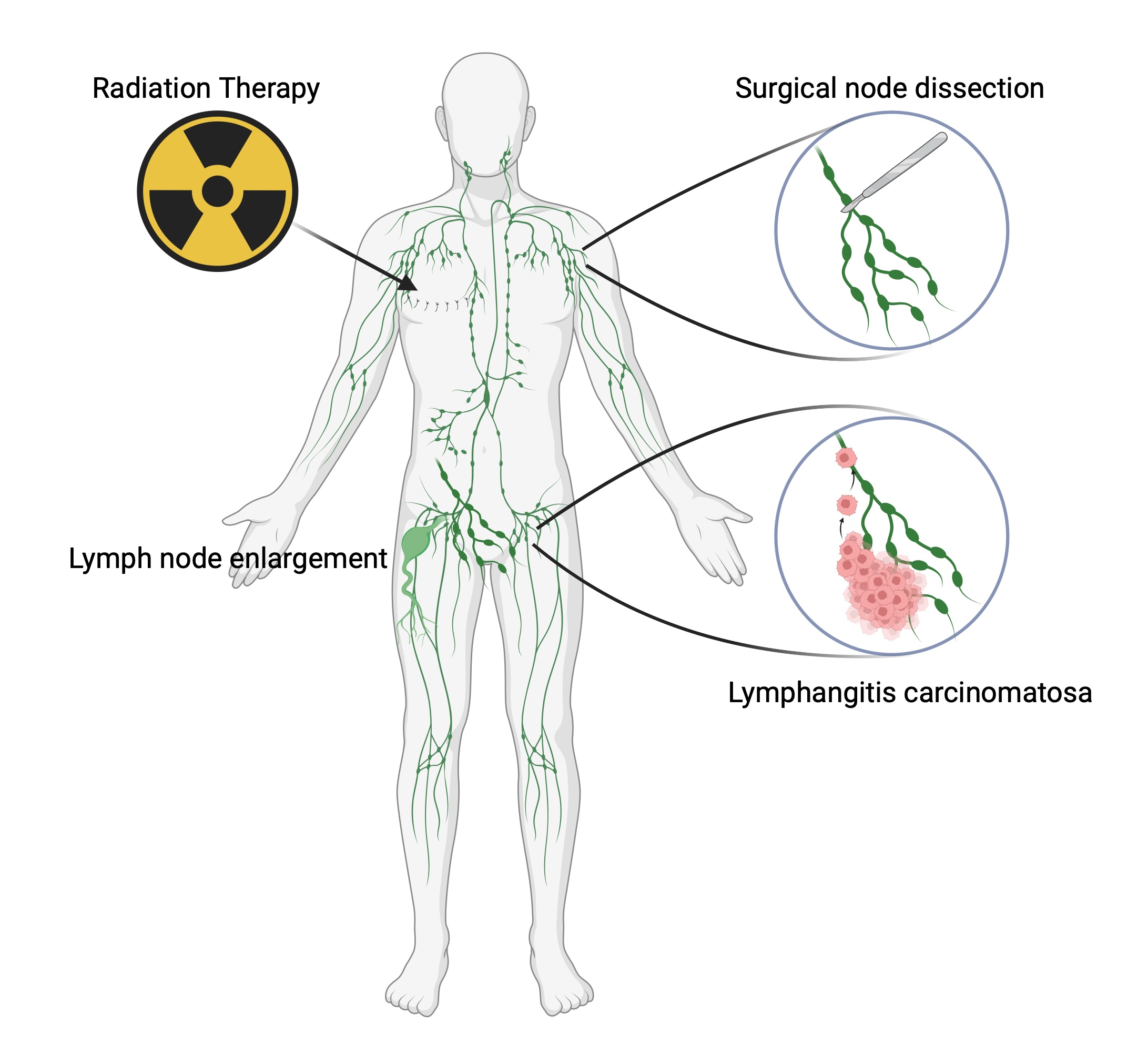

Supplement: tqaf029_Supplementary_Data [file tqaf029_supplementary_data.zip › tqaf029_Supplementary_Data/Sup 1.jpeg]

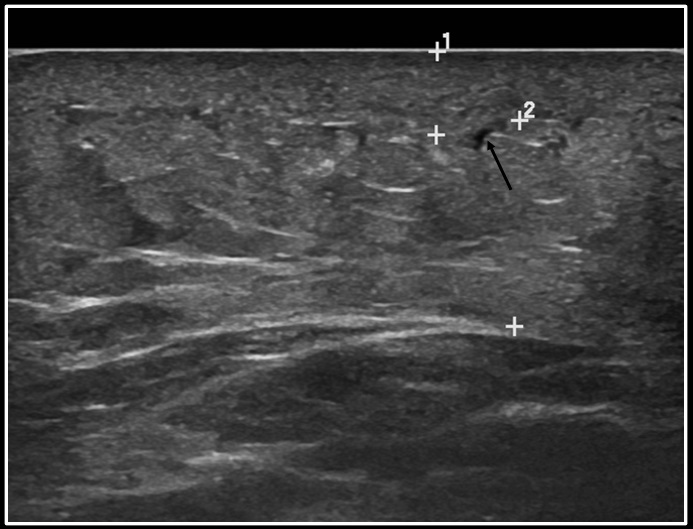

Supplement: tqaf029_Supplementary_Data [file tqaf029_supplementary_data.zip › tqaf029_Supplementary_Data/Sup 2.jpg]

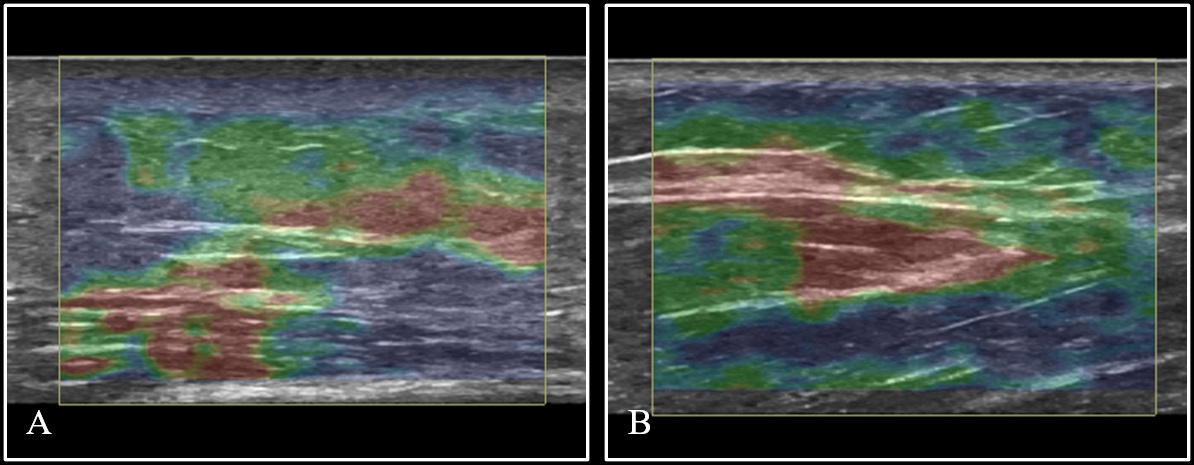

Supplement: tqaf029_Supplementary_Data [file tqaf029_supplementary_data.zip › tqaf029_Supplementary_Data/Sup 3.jpg]
